# Modeling Electronic Skin Response to Normal Distributed Force

**DOI:** 10.3390/s18020459

**Published:** 2018-02-03

**Authors:** Lucia Seminara

**Affiliations:** Department of Electrical, Electronics and Telecommunication Engineering and Naval Architecture, via Opera Pia 11A, University of Genoa, Genoa 16145, Italy; lucia.seminara@unige.it; Tel.: +39-10-353-2757

**Keywords:** PVDF sensors, electronic skin, skin modeling, solid mechanics

## Abstract

The reference electronic skin is a sensor array based on PVDF (Polyvinylidene fluoride) piezoelectric polymers, coupled to a rigid substrate and covered by an elastomer layer. It is first evaluated how a distributed normal force (Hertzian distribution) is transmitted to an extended PVDF sensor through the elastomer layer. A simplified approach based on Boussinesq’s half-space assumption is used to get a qualitative picture and extensive FEM simulations allow determination of the quantitative response for the actual finite elastomer layer. The ultimate use of the present model is to estimate the electrical sensor output from a measure of a basic mechanical action at the skin surface. However this requires that the PVDF piezoelectric coefficient be known a-priori. This was not the case in the present investigation. However, the numerical model has been used to fit experimental data from a real skin prototype and to estimate the sensor piezoelectric coefficient. It turned out that this value depends on the preload and decreases as a result of PVDF aging and fatigue. This framework contains all the fundamental ingredients of a fully predictive model, suggesting a number of future developments potentially useful for skin design and validation of the fabrication technology.

## 1. Introduction

Touch-sensitive electronic skin (e-skin) provides information on contact events occurring on its surface and can be used in a variety of contexts involving tactile interactions [1,2,3,4,5]. In recent decades, different tactile sensing technologies have been extensively developed especially for autonomous robotics [6,7,8]. However, there are a lack of models of the overall skin behavior, which would allow for better skin design and for the validation of the fabrication technology. Some interesting attempts in this direction have been made [9,10,11,12] e.g., to show how grasping information can be extracted from strain sensors beneath a compliant skin using simplified solid mechanics models and basic contact theory [13]. However, consideration of skin mechanics has been infrequent in the design and use of tactile sensors.

From a system perspective, e-skin includes stack-wise arrangements of functional and structural materials together with adequate interface electronics to read sensor signals [14]. In this paper, the reference architecture is a basic multilayer involving a rigid substrate, a Polyvinylidene fluoride (PVDF) piezoelectric polymer sensor array and an elastic layer on top for stress transmission and sensor protection. It is important to note that the whole theoretical analysis considers a single sensor skin. The design of a sensor array can be optimized based on the single sensor analysis, which studies how a distributed (Hertzian) force is transmitted to an extended sensor through an elastic layer. Indeed, needless to say, the presence of an array of sensors rather than a single sensor does not modify the analysis.

Although the first step for optimized skin design is characterizing the electromechanical and mechanical behaviors of e-skin functional (i.e., sensors) and structural (i.e., substrate and elastic cover) components, overall e-skin functioning is something different from that of its single building blocks. The skin is to be observed as a whole. How is contact force transmitted to each single extended sensor at the bottom of the elastic cover? Understanding what parameters affect the way mechanical information is transmitted to the sensor array is important to tailor e-skin design on application requirements. Relevant parameters in skin design are the size of sensors and their geometrical arrangement into the array as well as the properties of the protective layer, e.g., its thickness and compliance. To give an example, e-skin spatial resolution is determined by all these parameters. Furthermore, interesting sensing systems for the detection of tangential contact forces can be built starting from sensors that only measure pressure. The retrieval of tangential forces is permitted exclusively by the measurement of tensile stress immediately outside the compression region as measured by the sensor array [15]. However, for the skin to have this interesting functionality, the geometry of the sensor array and the properties of the protective layer need to satisfy certain requirements. Analogous considerations on the relation between skin compliance and tactile sensing abilities for a similar skin system based on strain sensors can be found in [13].

Moreover, building an electromechanical model of the artificial skin allows for validating skin fabrication technology and checking the whole process of assembling single components. To give some examples, for the piezoelectric polymer sensors to actually work in pure compression mode (common assumption), thus ensuring linearity between developed charge and received pressure, sensor bending is to be avoided. Partial bending of the sensors is naturally induced by not optimal integration procedures, for example inclusion of air bubbles into the coupling adhesive layer or bad sensor integration on the substrate, which is never ideally flat. Even assuming correct compression mode sensor behavior, the polarization process may not be homogeneous, inducing dispersion among sensors in the values of their d_33_ piezoelectric coefficients, which quantify pressure into charge conversion. Obtaining expected (modeled) behavior of the electrical response of each sensor to measured mechanical force at the skin surface proves that the whole fabrication process was successful, but for an acceptable dispersion in sensor functioning that can be considered intrinsic to the manufacturing process and handled by appropriate calibration.

In summary, the aim of illustrating the mechanism whereby mechanical information is transmitted to the sensor array is therefore twofold: first, to contribute developing a set of models for use in the electromechanical design of integrated sensing systems, secondly to pave the way for defining a set of tools for new skin technology validation.

The proposed model is static, but it may be likely extended to a dynamic contact, as long as its characteristic frequency is sufficiently far from the resonance frequencies of the system.

The rest of the paper is organized as follows. In the next section, materials and architecture of the reference skin system are first introduced. Subsequently, an electromechanical model of the electronic skin is presented, first studying the effect of (i) sensor size on sensor response to a single normal point force and of (ii) distributed normal force on point-like sensor response, and combining the two contributions to account for (iii) distributed normal force and extended sensor. In Section 3 the model is validated on a real skin prototype. Finally, the last section summarizes obtained results and open to future developments.

## 2. Materials and Methods

### 2.1. Electronic Skin Materials and Structure

**Figure 1:**
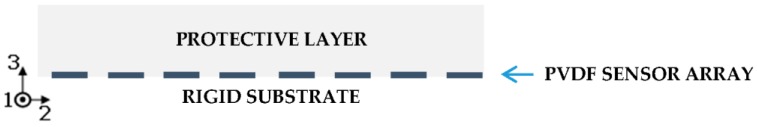
The PVDF sensor array is located on the bottom of the protective layer and integrated on a rigid substrate (side view).

In this paper, we consider a basic skin architecture that includes a rigid substrate, an array of PVDF sensors and a protective layer on top (Figure 1).

Functional materials in the form of a sensing array transform the mechanical information they receive into a set of electrical signals. PVDF piezoelectric polymers are an interesting choice for the sensing material, as they can measure dynamic contacts covering the whole frequency range of all human mechanoreceptors (<1 Hz–1 kHz) [16]. Due to the specific (orthotropic) PVDF symmetry and small film thickness (e.g., 100 µm for commercial films), when integrated on a rigid substrate PVDF sensors work in *thickness mode*, such that [17]:

*D*_3_ = d_33_*T*_3_, (1)

where, *D*_3_ is the charge density on the sensor surface, *T*_3_ is the normal stress component (i.e., the pressure acting on the bottom of the protective layer), and d_33_ is the piezoelectric coefficient. As PVDF sensors directly convert mechanical stimuli into charge, electronic circuits for data acquisition are based on charge amplifiers [18]. Equation (1) does not include the electric field across the PVDF sensor as it is assumed to be negligible due to the virtual ground at the operational amplifier inverting input. Hence, measuring the charge density on the sensor surface provides a direct measure of the normal stress acting on the sensor surface. Assuming circular sensors, the total sensor charge measured by the charge amplifier is given by:

(2) $$Q_{meas}=d_{33}\pi \;r_{T}{}^{2}{\bar{T}}_{3},$$

where $r_{T}$ is the sensor radius and ${\bar{T}}_{3}$ is the normal stress component *T*_3_ averaged over a single extended sensor.

The protective layer, which is usually polymer-based (e.g., PDMS), implements a mechanical filtering of the tactile stimulus applied at the skin surface and distributes the mechanical stimulus onto the sensor array below. The thickness of the soft layer is a very important parameter in the design of a flexible skin. In the case of the present study—where PVDF is placed at the device bottom and works in thickness mode—thicker layer means weaker sensor response signals. However, a thicker elastomer layer exalts its surface deflection under contact forces and warrants more accuracy in the measurement of changes of curvature if sensors are reduced to tiny compliant strips and placed near the outer elastomer surface.

### 2.2. Electronic Skin Model

Equation (2) relates PVDF sensor output (i.e., the measured charge *Q_meas_*) to the normal stress component *T*_3_ averaged over the sensor (i.e., ${\bar{T}}_{3}$). Scope of the following two sections is to retrieve the average normal stress component ${\bar{T}}_{3}$ transmitted to a single extended tactile sensor as a function of the normal force *F*_3_ applied at the surface of the skin protective layer. This would allow to estimate the sensor charge *Q_meas_* (output) as a function of the normal force *F*_3_ (input), which leads to an estimation of the system transfer function FRF, defined as the ratio between the Fourier transforms of the output charge and of the input force. In this paper, we only focus on normal contact forces.

Basic assumption of the proposed model (which can be relaxed in future steps) is that the e-skin protective layer can be treated as an incompressible elastic medium (Poisson ratio sufficiently close to 0.5). This is appropriate for an elastomer (*ν* = 0.48), but not for other materials (e.g., a foam).

In the present section we make an additional major assumption: we assume that our system, namely an elastic medium consisting of a layer with finite thickness, is part of an elastic half-medium bounded by the free surface on which the external force is applied. As shown below, this assumption is quite useful, allowing for an analytical solution of the mathematical problem in closed form, based on Boussinesq’s classical solution [19].

This approach provides a qualitative picture of the response of the e-skin to external forces that lets the relevant physical parameters emerge naturally from the analysis. However, the price paid is the inability of the approach to account for the actually finite thickness of the elastomer as well as to impose the boundary condition at the rigid substrate. In the next section we then relax the latter assumption and solve the problem numerically for the actual physical configuration. This will allow us to obtain a quantitatively more reliable picture suitable for experimental validation.

### 2.3. Simplified Analysis

#### 2.3.1. Effect of Sensor Size on Sensor Response to a Single Normal Point Force

**Figure 2:**
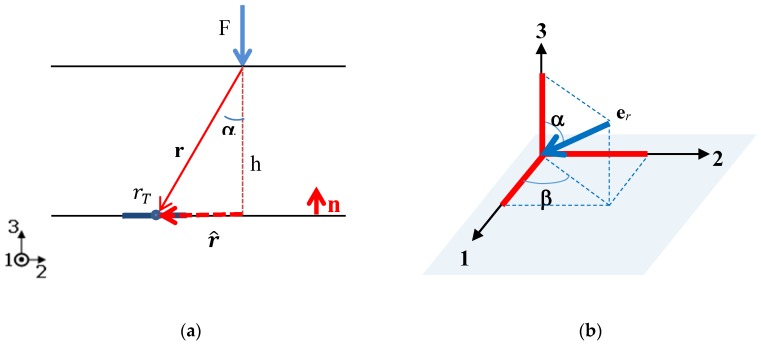
(**a**) The PVDF sensor (radius $r_{T}$) is located on the bottom of the elastic cover of thickness *h* and a normal point force is applied on the outer surface (view side). (**b**) **e***_r_* is represented in a spherical coordinate system.

Consider a single sensor of radius $r_{T}$ at a fixed position on a rigid surface (not necessarily plane) with unit normal vector **n**. Assume a point force F is applied at a given position on the outer surface of the layer. The surface is coated with an elastomer layer of constant thickness *h* (Figure 2a). A stress field is then generated in the elastomer and transmitted to the sensor. Let **T** denote the stress tensor. The stress vector acting on the sensor reads **n**·**T**. This stress response can be conveyed into an appropriate circuit and retrieved by an electronic device. For example, PVDF sensors directly convert the *T*_3_ stress component into charge, as indicated by Equation (1).

In order to determine the relation between a point load **F** applied on the outer surface and the stress at a given point inside the cover layer, we may get advantage of the solution of the so-called Boussinesq’s problem [19]. This problem considers an elastic half-medium bounded by a surface on which a point force is applied. For such a configuration the stress field determined by Boussinesq reads:

(3) $$T=\frac{3}{2\pi }\frac{F\cdot e_{r}}{r^{2}}e_{r}⊗e_{r},$$

where **r** is the radial distance of the generic point of the medium from the application point of the load **F**, all bold-faced symbols represent tensors or vectors, **e***_k_* is the unit vector in the *k*-direction and ⊗ is the symbol of tensor product.

Let us next assume that Equation (3) may be applied to our configuration, approximating our finite thickness layer with Boussinesq’s semi-infinite medium. Hence, the stress tensor at the center of the sensor will be evaluated by Equation (3), with **r** separation vector of the force application point from the center of the sensor area. The advantage of Equation (3) is its simplicity, as the stress is uniaxial in the radial direction, and its independence of elastic parameters.

In this paper, we develop Equation (3) considering the sole *T*_3_ stress component on the bottom of the elastic cover of thickness *h*, as received by a PVDF sensor working in thickness mode Equation (1). Letting $\hat{r}$ be the radial distance of the point where the force is applied from the sensor center projected on the outer surface (see Figure 2a), we have r^2^ = $\hat{r}$^2^ + *h*^2^ and **e***_r_* = *sin*α (**e**_1_ *cos*β + **e**_2_ *sin*β) − **e**_3_ *cos*α (see Figure 2b), where *cos*α = *h*/*r*, *sin*α = $\hat{r}$/*r*.

(4) $$T_{3}=\frac{3}{2\pi }\frac{h^{2}}{{\left({\hat{r}}^{2}+h^{2}\right)}^{5/2}}\left\{\left(F_{1}cos\beta +F_{2}sin\beta \right)\hat{r}-F_{3}h\right\},$$

For a vertical contact force *F*_3_ (*F*_1_ = *F*_2_ = 0), Equation (4) reduces to

(5) $$T_{3}=-\frac{3}{2\pi }\frac{F_{3}h^{3}}{{\left({\hat{r}}^{2}+h^{2}\right)}^{5/2}}=-\frac{3}{2\pi }\frac{F_{3}}{h^{2}}\frac{1}{{\left(\lambda^{2}+1\right)}^{5/2}},$$

where $\lambda =\frac{\hat{r}}{h}$.

**Figure 3:**
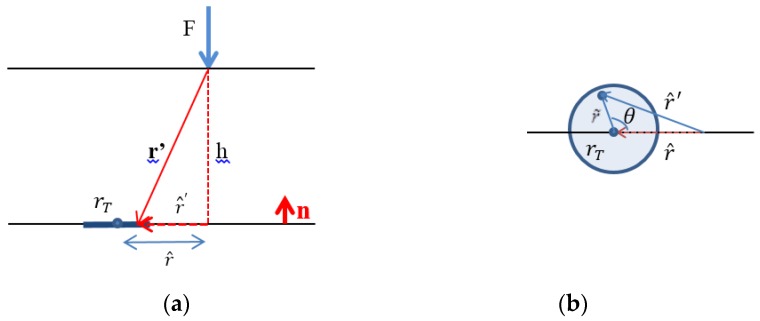
(**a**) View side, (**b**) Top side.

As the sensor is not point-like, it is convenient to average the stress over the surface of the sensor. With the notations of Figure 3 we find:

(6) $${\bar{T}}_{3}=-\frac{3}{2\pi }\frac{F_{3}}{h^{2}}\frac{1}{\pi {\left(\frac{r_{T}}{h}\right)}^{2}}\int_{0}^{2\pi }\int_{0}^{\frac{r_{T}}{h}}\frac{\tilde{\lambda }d\tilde{\lambda }d\theta }{{\left({\tilde{\lambda }}^{2}+\lambda^{2}-2\tilde{\lambda }\lambda cos\theta +1\right)}^{5/2}}=-\frac{3}{2\pi }\frac{F_{3}}{h^{2}}\gamma \;(\frac{\hat{r}}{h},\frac{r_{T}}{h}),$$

As ${\hat{r}\prime }^{2}={\tilde{r}}^{2}+{\hat{r}}^{2}-2\tilde{r}\hat{r}cos\theta$, λ = $\frac{\hat{r}}{h}$ and $\frac{\tilde{r}}{h}$ is defined as $\tilde{\lambda }$.

In Equation (6), the $\gamma \;(\frac{\hat{r}}{h},\frac{r_{T}}{h})$ coefficient includes the double integral, which can be solved numerically as a function of $\frac{\hat{r}}{h}$, i.e., the dimensionless distance of the sensor center from the projection of the force application point on the sensor plane. Note that this is the first important result of the analysis: indeed, γ is a measure of how the normal point force *F_3_* is transmitted to the sensor through the elastic layer leading to an average normal stress ${\bar{T}}_{3}$ acting on the sensor.

**Figure 4:**
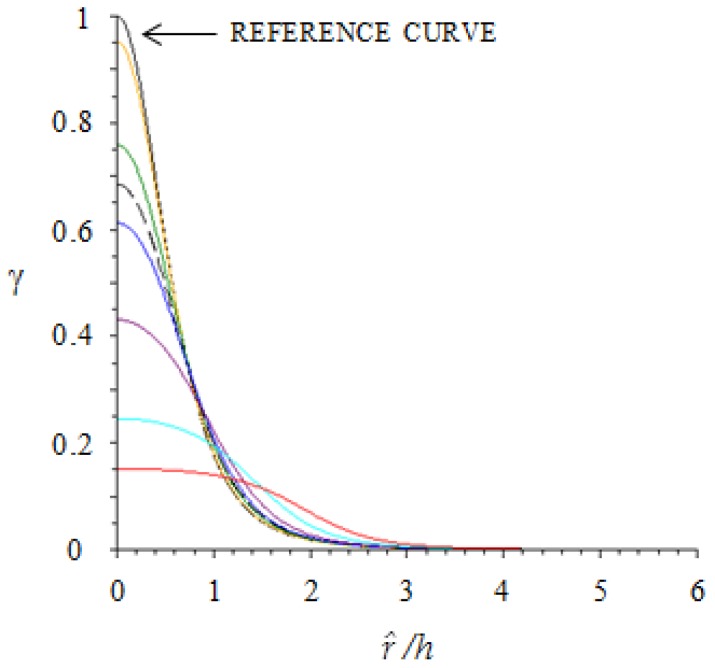
The proportionality coefficient γ between average normal stress ${\bar{T}}_{3}$ on the sensor and normal point force *F_3_* (see Equation (6)) is plotted versus the distance $\hat{r}$ from sensor center (see Figure 2 for notations). Different curves are associated with different sensor sizes as measured by the dimensionless parameter $\frac{r_{T}}{h}$ (*yellow* $\frac{r_{T}}{h}=0.2$; *green* $\frac{r_{T}}{h}=0.5$; *black dotted* $\frac{r_{T}}{h}=0.6$; *blue* $\frac{r_{T}}{h}=0.7$ ; *magenta* $\frac{r_{T}}{h}=1$; *light blue* $\frac{r_{T}}{h}=1.5$; *red* $\frac{r_{T}}{h}=2$).

In Figure 4, the dependence of $\gamma \;(\frac{\hat{r}}{h},\frac{r_{T}}{h})$ on $\frac{\hat{r}}{h}$ is plotted for different skin designs, measured by the ratio of sensor radius to the thickness of the layer, $\frac{r_{T}}{h}$. The reference black curve corresponds to the case of “point-like” sensor ($r_{T}=0$). The color legend is included in the figure caption.

From an analysis of Figure 4, it follows that, for sufficiently small values of $\frac{r_{T}}{h}$ (say 0.2, yellow curve), the problem can be treated as if the sensor was point-like. In the other cases, the problem can still be treated as if the sensor was point-like provided that the force is located at a sufficient distance from the sensor center. For example, if $\frac{r_{T}}{h}=1$ (magenta curve) in order to treat the sensor as point-like the force is to be located at a distance that is equal/larger than the sensor radius.

Figure 4 is an important first achievement of this analysis, as it can be used as a practical tool for skin design. As a matter of fact, it allows one to optimize the system geometry (*r_T_*/*h* and sensor pitch) to achieve a desired skin resolution, which is related to the spatial concentration of the mechanical stress information around a single sensor.

#### 2.3.2. Effect of Normal Force Distributed Over Circular Contact Area on Point-Like Sensor Response

**Figure 5:**
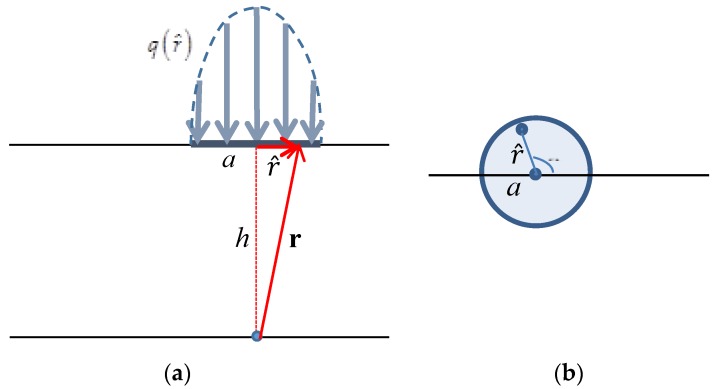
Normal force distributed over circular contact area with Hertzian pressure distribution. (**a**) View side, (**b**) Top side.

Consider normal force acting on a circular contact area of radius *a* with Hertzian pressure distribution $q\left(\hat{r}\right)$ [20], which realistically describes normal contact by a rigid spherical indenter (Figure 5):

(7) $$q\left(\hat{r}\right)=q_{0}{\left\{1-\frac{{\hat{r}}^{2}}{a^{2}}\right\}}^{\frac{1}{2}},$$

The reader is warned that, unlike in Section 2.3.1, here $\hat{r}$ denotes a radial coordinate with origin at the center of the contact area (see sketch in Figure 5).

The overall contact force is the result of integrating $q\left(\hat{r}\right)$ over the contact area (Figure 5b)

(8) $$F_{3}=\int_{0}^{a}q(\hat{r})\hat{r}d\hat{r}\int_{0}^{2\pi }d\theta ,$$

Using Equation (7) into Equation (8) allows for retrieving $q_{0}$ as follows:

(9) $$q_{0}=\frac{3}{2\pi }\frac{F_{3}}{a^{2}},$$

The radius *a* of the imprint is related to the applied load *F*_3_ by the equation [20]:

(10) $$a^{3}=\frac{3F_{3}R}{4E^{*}}=\frac{3F_{3}R}{4E}(1-\nu^{2}),$$

where *R* is the radius of the spherical indenter, *E* is the elastic modulus of the protective layer and *ν* its Poisson coefficient.

In the following, we describe the effect of this distributed force on a point-like sensor, which is aligned with the contact center. Considering other sensor positions is far from the scopes of this paper.

Having in mind that Equation (3) is the Green function of the problem, the normal stress *T*_3_ generated on a point-like sensor located at a depth *h* below the center of the contact area can be calculated by:

(11) $$T_{3}=-\frac{3}{2\pi }\int_{0}^{2\pi }d\theta \int_{0}^{a}\frac{q\left(\hat{r}\right)\hat{r}h^{3}d\hat{r}}{{\left({\hat{r}}^{2}{+h}^{2}\right)}^{5/2}}=-\frac{3}{2\pi }\int_{0}^{2\pi }d\theta \int_{0}^{a}\frac{q_{0}{\left\{1-\frac{{\hat{r}}^{2}}{a^{2}}\right\}}^{\frac{1}{2}}\hat{r}h^{3}d\hat{r}}{{\left({\hat{r}}^{2}{+h}^{2}\right)}^{5/2}},$$

By introducing new integration variable $\lambda =\frac{\hat{r}}{h}$, defining $a*=\frac{a}{h}$ and using Equation (9) for $q_{0}$ we get:

(12) $$T_{3}=-\frac{3}{2\pi }\frac{F_{3}}{h^{2}}\frac{3}{2\pi a^{*}{}^{2}}\int_{0}^{2\pi }d\theta \int_{0}^{a*}\frac{{\left\{1-\frac{\lambda^{2}}{a^{*}{}^{2}}\right\}}^{\frac{1}{2}}\;\lambda \;d\lambda }{{\left(\lambda^{2}+1\right)}^{5/2}}=-\frac{3}{2\pi }\frac{F_{3}}{h^{2}}\;\delta (\frac{a}{h}),$$

**Figure 6:**
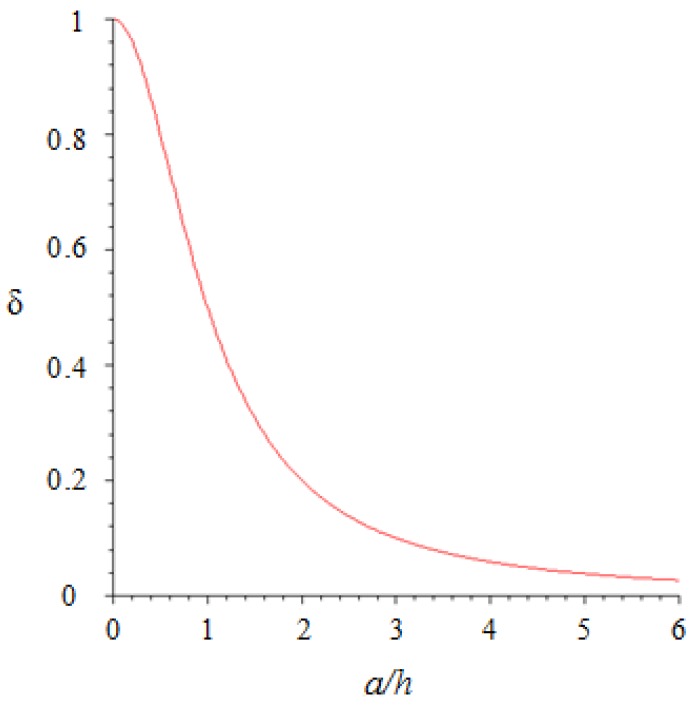
The proportionality coefficient δ between normal stress *T*_3_ on the sensor and overall contact force *F_3_* (see Equation (12)) is plotted versus the imprint radius *a* (contact size) scaled by the elastomer thickness *h* (see Figure 5 for notations). Note that the point-like sensor is aligned with the contact center.

In Equation (12), the $\delta \;(\frac{a}{h})$ coefficient includes the double integral, which can be solved numerically as a function of $\frac{a}{h}$, i.e., the contact radius (depending on *F*_3_ through Equation (10)) normalized to the layer thickness. Note that this is the second important result of the analysis: indeed, δ is a measure of how the force *F*_3_ (distributed with Hertzian pressure distribution $q\left(\hat{r}\right)$) is transmitted to the point sensor through the elastic layer leading to the stress *T*_3_ on the sensor. In Figure 6, δ  is plotted as a function of $\frac{a}{h}$.

#### 2.3.3. Combination of the Two Contributions: Effect of Distributed Normal Force on the Response of an Extended Sensor

**Figure 7:**
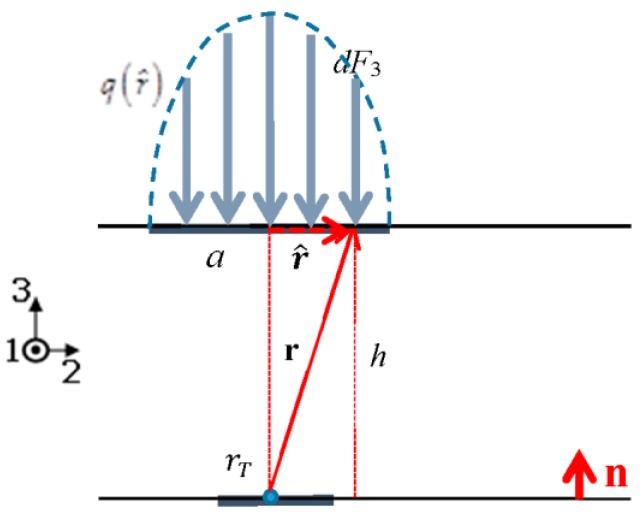
The extended PVDF sensor is located on the bottom of the elastic cover of thickness *h* and a distributed normal force applied on the outer surface is aligned with the sensor center.

Starting from the case study described in Section 2.3.1, if the force is distributed with Hertzian pressure distribution (Figure 7), Equation (6) can be written as:

(13) $${T‾}_{3}=-\frac{3}{2\pi }\frac{1}{h^{2}}\int_{contactAREA}{dF}_{3}(\frac{\hat{r}}{h},\theta )\;\gamma \;(\frac{\hat{r}}{h},\frac{r_{T}}{h})=-\frac{3}{2\pi }\frac{1}{h^{2}}\int_{0}^{a}\frac{3}{2\pi }\frac{F_{3}}{a^{2}}{\left\{1-\frac{{\hat{r}}^{2}}{a^{2}}\right\}}^{\frac{1}{2}}\;\gamma \;(\frac{\hat{r}}{h},\frac{r_{T}}{h})\hat{r}d\hat{r}\int_{0}^{2\pi }d\theta ,$$

Note that the function $\gamma \;(\frac{\hat{r}}{h},\frac{r_{T}}{h})$, which weighs the contribution of each point force *dF_3_* to the average normal stress *T_3_* is a function of the radial distance of the point force *dF*_3_ from the projection of the sensor center on the outer surface. Recall that this distance coincides with the radial coordinate $\hat{r}$ employed to integrate over the contact area. Hence, the function γ must be included inside the integral, as shown by Equation (13).

By introducing new integration variable $\lambda =\frac{\hat{r}}{h}$ and defining $a*=\frac{a}{h}$, we get:

(14) $${\bar{T}}_{3}=-\frac{3}{2\pi }\frac{F_{3}}{h^{2}}\frac{3}{2\pi a^{*}{}^{2}}\int_{0}^{2\pi }d\theta \int_{0}^{a*}{\left\{1-\frac{\lambda^{2}}{a^{*}{}^{2}}\right\}}^{\frac{1}{2}}\;\lambda \;\;\gamma \;(\lambda \;,\frac{r_{T}}{h})\;d\lambda =-\frac{3}{2\pi }\frac{F_{3}}{h^{2}}\sigma (\frac{a}{h}\;,\frac{r_{T}}{h})\;,$$

As above, for each chosen $\frac{r_{T}}{h}$, $\sigma \;(\frac{a}{h},\frac{r_{T}}{h})$ includes the double integral that must be solved numerically as a function of $\frac{a}{h}$, i.e., the contact radius scaled by the layer thickness. This is the most important result of this analysis: indeed, σ is a measure of how the normal distributed force *F_3_* is transmitted to the sensor through the elastic layer leading to an average normal stress ${\bar{T}}_{3}$ acting on the sensor.

**Figure 8:**
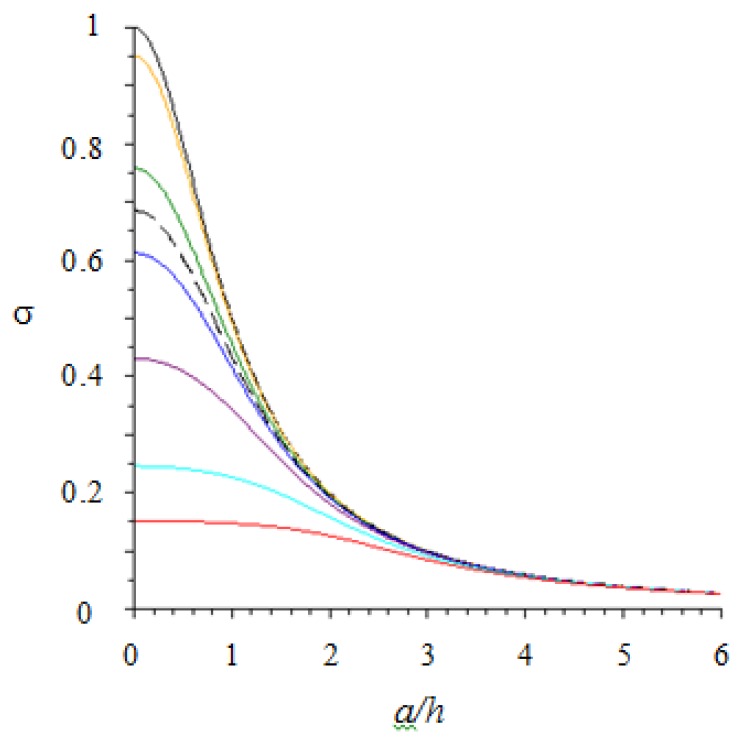
The proportionality coefficient σ between average normal stress ${\bar{T}}_{3}$ on the sensor and overall (Hertzian) contact force *F_3_* (see Equation (14)) is plotted versus the imprint radius *a* (contact size) scaled by the elastomer thickness *h* (see Figure 7 for notations). Note that the applied force is centered on the sensor. Different curves are associated with different sensor sizes as measured by the dimensionless parameter $\frac{r_{T}}{h}$ (*yellow* $\frac{r_{T}}{h}=0.2$; *green* $\frac{r_{T}}{h}=0.5$; *black dotted* $\frac{r_{T}}{h}=0.6$—***case study reported in the experimental session***; *blue* $\frac{r_{T}}{h}=0.7$; *magenta* $\frac{r_{T}}{h}=1$; *light blue* $\frac{r_{T}}{h}=1.5$; *red* $\frac{r_{T}}{h}=2$).

In Figure 8, we plot $\sigma \;(\frac{a}{h},\frac{r_{T}}{h})$ as a function of $\frac{a}{h}$. The reference black curve corresponds to the case of distributed force centered on point-like sensor and, as expected, it coincides with the function $\delta \;(\frac{a}{h})$. On the other hand, in the case of normal point force aligned with the sensor center (*a* = 0), we find $\sigma (0,\frac{r_{T}}{h})\;=\gamma (0,\frac{r_{T}}{h})\;$ (the intercepts of $\sigma (\frac{a}{h},\frac{r_{T}}{h})\;\;$ and $\gamma (\frac{a}{h},\frac{r_{T}}{h})$ on the *y*-axis are the same).

From an analysis of Figure 8, it follows that, for sufficiently small values of $\frac{r_{T}}{h}$ (say 0.2, yellow curve), the problem can be treated as if the sensor was point-like. In the other cases, the problem can still be treated as if the sensor was point-like provided the contact radius is sufficiently large. For example, if $\frac{r_{T}}{h}=1$ (magenta curve) in order to treat the sensor as point-like the force is to be distributed over a contact area with radius that is at least twice the layer thickness.

Until this point, the specific type of sensor did not come into play. The previous analysis is completely independent of the specific sensor type and only describes the role of the elastomer layer in stress transmission. The specific sensor type (i.e., PVDF piezoelectric polymer) is now included into the picture. Using Equation (14), the charge response of a single extended sensor to distributed normal force centered on the sensor can be estimated as:

(15) $$Q_{3}=\pi r_{T}^{2}d_{33}{\bar{T}}_{3}=-d_{33}\frac{3}{2}{\left(\frac{r_{T}}{h}\right)}^{2}\;\sigma \;(\frac{a}{h},\frac{r_{T}}{h})\;\;F_{3}$$

For a given skin geometry Equation (15) allows one to calculate the effective piezoelectric coefficient d_33_ of each PVDF sensor, once the charge *Q_3_* and the force *F_3_* have been measured. Comparison with the expected value of d_33_ [21] allows one to validate sensor functioning and skin technology.

### 2.4. FEM Simulations

**Figure 9:**
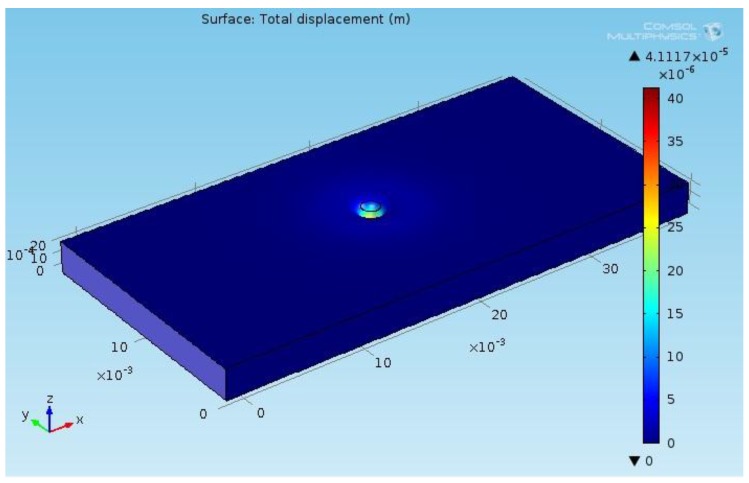
An example of FEM simulation results (total displacement is shown). Parameters: *F*_3_ = 2 N, *R* = 10 mm, *E* = 16 MPa, *ν* = 0.5, *r_T_* = 1 mm. Elastomer size: thickness *h* = 2.5 mm, length l = 40 mm, width b = 20 mm.

We now remove the severe assumption whereby the elastomer may be modeled as an elastic half-medium. In other words, we consider an elastic incompressible medium consisting of a layer of finite thickness h, length l and width b (Figure 9). Length and width of the layer have been chosen arbitrarily, with the sole requirement of the elastomer sides being distant “enough” from the considered sensor such to justify the assumption that the lateral boundaries do not affect the stress field acting on the sensor significantly.

The free surface is assumed to be subject to an external Hertzian pressure distribution Equation (7), with *q*_0_ small enough to lead to small amplitude deformations. The lower boundary is assumed to be rigid, while the side walls are free. The solution of the problem is sought numerically, with the help of the code COMSOL Multiphysics. Figure 9 illustrates an example of the results of FEM simulations of the elastomer subject to a normal force distributed over circular contact area (black line, radius *a* Equation (10)) with Hertzian pressure distribution. The employed value for the elastomer modulus E is the result of experimental characterization of the elastic layer of the reference e-skin as discussed in Section 3.2.1.

**Figure 10:**
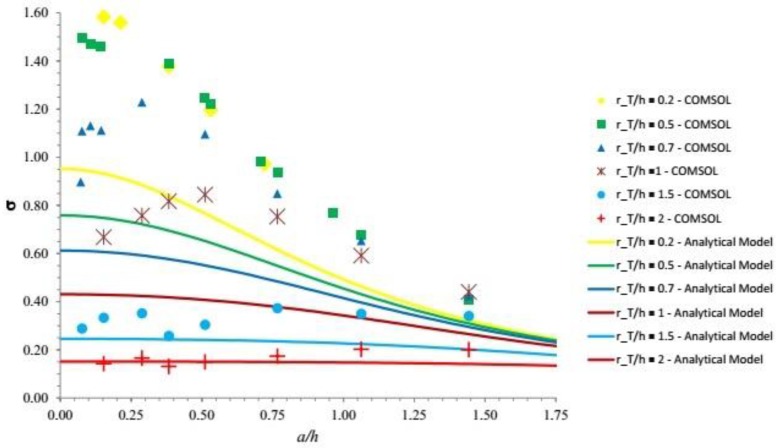
A comparison between results for σ as a function of *a*/*h* for different values of *r_T_*/*h* (see legend), as obtained by Boussinesq’s analytical model (see Figure 8) and the numerical COMSOL simulations for the finite case.

Simulations have been initially performed by assigning fixed values of *ν* and *E* (see Section 3.2.1). Moreover, for any given *r_T_*/*h*, the value of *a*/*h* has been changed by arbitrarily playing with the two parameters *F*_3_ and *R* in Equation (10). The output of the simulation is reported in Figure 10, where it is compared with the simplified analytical solution based on Boussinesq’s approach.

Figure 10 shows that, under the conditions of the configuration investigated in numerical simulations, the numerical output follows a similar qualitative trend but it differs quantitatively from the analytical results discussed in the previous section. This is mainly due to the boundary condition imposed by the rigid substrate. However, note that if the substrate is moved at a depth higher than h, the two solutions do approach each other. This has been verified, performing further simulations where the substrate was set at depths of 10 mm and 30 mm. It turns out that—for *r_T_*/*h* = 0.2 and *a*/*h* = 0.289—the relative error of the analytical solution with respect to the numerical one decreases from about 40% to about 10% at 10 mm depth and 1% at 30 mm depth. This suggests that for sensors embedded into a thick protective layer the analytical solution would be quite appropriate.

It is also important to note that Figure 10 displays apparently irregular oscillations of the response for any given *r_T_*/*h*. This has prompted us to identify what causes these oscillations. Indeed, at a more careful examination, it turns out that, in the finite case, σ depends on an additional parameter, besides *a*/*h* and *r_T_*/*h*. This may be readily appreciated noting that, on physical ground, ignoring the effects of the sidewalls, the response of the system may be assumed to depend on the following dimensional quantities: *h*, *r_T_*, *E*, *F* and *R*. With the help of simple dimensional arguments, one may then envisage the following dimensionless relationship:

σ = σ (*R*/*h*, *r_T_*/*h*, *F*/(*R*^2^ × *E*)) (16)

**Figure 11:**
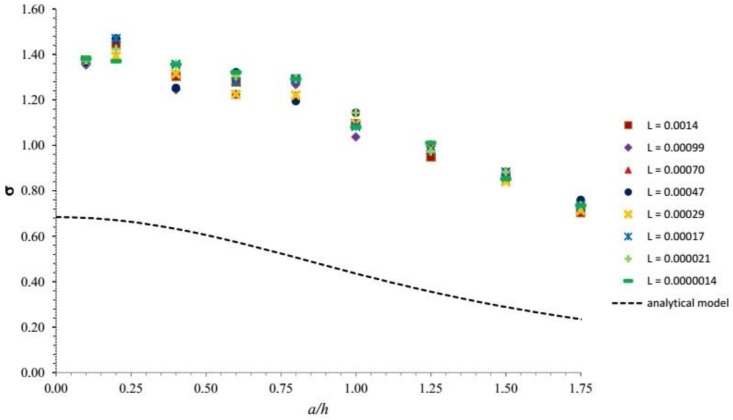
A comparison between Boussinesq’s analytical model for the half-space (dotted line) and the numerical COMSOL simulations for the finite case (markers) is reported for *r_T_*/*h* = 0.6, corresponding to the geometry of the e-skin prototype employed in the experimental section. As in Figure 8 and Figure 10, σ vs. *a/h* is plotted. The role of the parameter L is included and confirms that distinct curves are associated with different values of L.

Note that, in view of Equation (10), the parameter *R*/*h* is equivalent to the parameter *a*/*h* previously used. Below, we denote the additional parameter *F*/(*R*^2^ × *E*) by L. The above argument suggests that the plot of Figure 10 must be modified such that each line corresponding to given *r_T_*/*h* be replaced by a strip of lines each associated with the same value of *r_T_*/*h* but a distinct value of L. The output of the simulations is therefore organized including the dependence of σ on the parameter L. Figure 11 illustrates the results for *r_T_*/*h* = 0.6, which corresponds to the geometry of the real e-skin prototype employed for experimental tests presented in the following section. Note that the figure confirms that distinct curves are associated with different values of L. Similar analysis can be extended to all values of *r_T_*/*h* and analogous strips of lines would be obtained. However, in Figure 11 we have restricted ourselves to a range of values of the parameter L of direct physical relevance for the tactile application.

## 3. Experimental Results and Discussion

A series of tests has been finally performed on an electronic skin based on arrays of PVDF transducers, applying a normal contact force on the e-skin surface by a rigid spherical indenter (Hertzian pressure distribution). For the sake of convenience, the normal contact force was aligned with the sensor center, hence a distinct run was needed for each sensor. Experiments allowed measurement of the system response function FRF. We recall that FRF corresponds to the ratio between the Fourier transform of the output charge and that of the input force.

Of course, FRF can also be predicted using the theoretical model. However, the latter prediction depends on the effective piezoelectric coefficient d_33_ of each PVDF sensor, a quantity that was a priori unknown in the present investigation. Hence, comparison between theoretical predictions and experimental observations has been employed to infer the value of the effective piezoelectric coefficient d_33_ that leads to best fit. In other words, at the present stage this model may be described as post-dictive. It will become pre-dictive once the effective piezoelectric coefficient d_33_ will be known a-priori.

A delicate issue arises because the PVDF piezoelectric polymer does not read static information, i.e., the model can only be used with dynamic contacts. A dynamic contact has been indeed employed, as explained below, relying on the expectation that such an approach is safe provided that the forcing frequencies fall outside the range of any significant resonance. Resonances barely derive from the stimulated sensor, as they would result from longitudinal waves which have much higher characteristic frequencies (≈100 kHz) than those related to contact (<1 Hz–1 kHz). The order of magnitude of resonance frequencies *f* associated with longitudinal waves can be calculated from $f=\frac{v}{\lambda }$, where v is the speed of sound in the elastomer layer (≈1000 m/s [22]) and $\frac{\lambda }{4}$ corresponds to the thickness of the elastomer layer (*h* = 2.5 mm). Resonances may also derive from the sensor itself, but we have previously proven [21] that its charge-force transfer function (d_33_(ω)) is approximately flat in the frequency range of interest for the tactile application (say, 1 Hz–1000 Hz). This notwithstanding, resonances may derive from a variety of additional causes (e.g., movable contacts, contact surface asperities, motor-induced vibrations), which cannot be a-priori controlled. The ultimate solution is then to identify a flat zone in the system response function and perform experiments only in that frequency range.

### 3.1. Experimental Setup

As mentioned in the introduction, the tactile sensing system we are considering basically consists of an array of stress sensors fixed on a rigid substrate and coated with an elastomer layer. An example of a real device of this kind, based on an array of piezoelectric PVDF sensors, is illustrated in [23]. This e-skin prototype integrates 64 ad-hoc screen-printed electrodes and tracks on both sides of a commercial piezoelectric polymer film (Measurement Specialties Inc., Hampton, VA, USA, http://www.meas-spec.com/default.aspx). Sensor radius equals 1.5 mm and pitch between adjacent sensors is 8 mm. As the flexible skin has been glued on a rigid substrate, the tactile stimulus applied on the outer surface of the elastomer (thickness *h* = 2.5 mm) is received by the sensors as a distribution of normal stresses (*T*_3_), which is converted into charge.

**Figure 12:**
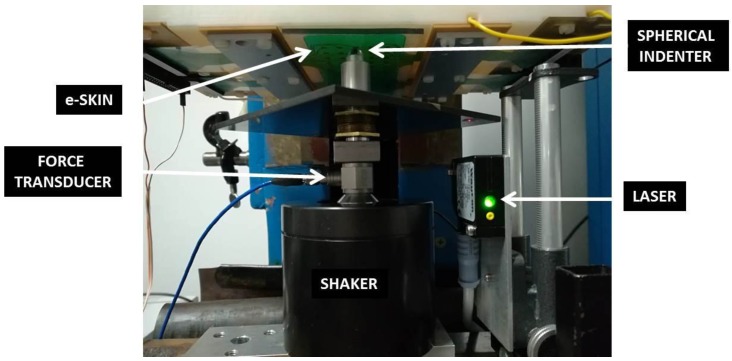
Experimental equipment for e-skin validation.

The experimental setup (Figure 12) is a slightly different version of that described in [21].

It consists of a rigid frame with a lower fixed plate to which an electro-mechanical shaker (Brüel and Kjaer, Nærum, Denmark, Minishaker Type 4810 with Power Amplifier Type 2706) is assembled. A piezoelectric force transducer (Model 208C01, PCB Piezotronics, Depew, NY, USA) is fixed to the moving head of the shaker. The e-skin sample is faced down side and it is mechanically stimulated by the rigid spherical indenter coupled to the mini-shaker. The loading chain is formed, accordingly, by: shaker, force transducer, spherical indenter, and skin sample. All these elements have to be aligned before any test, for the indenter to be centered on a specific sensor. The mini-shaker is controlled through a graphical user interface (GUI) developed with NI LabVIEW on the host PC and NI DAQ data acquisition board is used. A swept sine signal is fed into the shaker (settable parameters: start and end frequencies, number of steps, amplitude). The output signals of sensor charge (response) and force transducer (stimulus) are continuously acquired by a 4-channel PCB Sensor Signal Conditioner 482C54 (ICP and charge mode) and processed in frequency (i.e., their Fourier transforms are calculated by LabVIEW software) to give the system response function FRF at each frequency step. Before running each test, a preload is applied to guarantee indenter-skin contact during the whole mechanical stimulation. It is this preload which is responsible for determining the contact radius *a* Equation (10), as for all tests the amplitude of the dynamic oscillation is maintained low enough not to affect significantly the contact area. This preload is controlled using a laser to measure the displacement of the flat plate coupled to the shaker and using shaker displacement-force calibration curves.

### 3.2. Results

In view of the analysis discussed in Section 2.4, it is appropriate to employ only the full numerical solution of the elastic problem for the actual e-skin prototype to fit experimental data.

#### 3.2.1. Characterization of the Elastic Properties of the Elastomer

**Figure 13:**
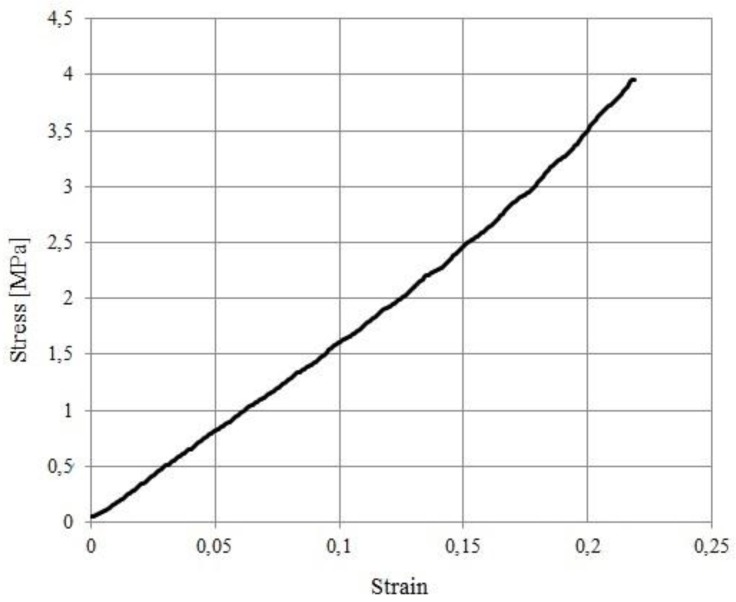
The stress-strain curve for the elastomer sample in a compression test.

The compressive Young’s modulus of the PDMS elastic layer was measured using an electromechanical machine Zwick/Roell Z0.5 (maximum load 500 N, operating with TestXpert II software), on a cylindrical sample of 4 mm diameter and 2.5 mm length (Figure 13). The test was performed at a speed of 10 mm/min, in the displacement control mode.

The elastic Young’s modulus of the elastomer has been calculated as the slope of the first linear portion of the curve in Figure 13 and it corresponds to 16 [MPa]. This value has been employed for all numerical models in this paper. Note that non-linear elastomer behavior starts at stress values about 2 [MPa].

#### 3.2.2. Frequency Selection

The first issue is identifying the appropriate range of frequencies of the mechanical stimulus to validate the electronic skin. As said in the introduction to Section 3, this dynamic approach proves safe provided that the range of forcing frequencies falls outside of any significant system resonance.

**Figure 14:**
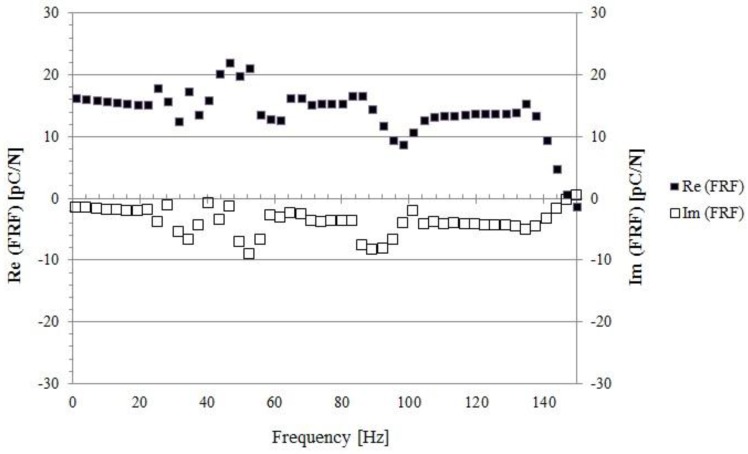
The system response function FRF (Re and Im) over the range 1–150 Hz is shown.

First tests have been performed to record the system response function (FRF) experimentally over the whole frequency range (1 Hz–1000 Hz). It turns out that: (i) resonances do exist and there characteristic frequencies depend on the preload and the size of the indenter; (ii) a fairly wide range of frequencies exists, where no significant resonance is detected; (iii) the imaginary part of the response function, which accounts for any viscoelastic component of the response, is roughly an order of magnitude smaller than the real (elastic) part. The latter statement is clarified in Figure 14, where the system response function is plotted in the non-resonant range (roughly 1–130 Hz).

Based on these results, hereafter the imaginary part of the response will be ignored and Re will be removed from the notation. In other words, the system is treated as purely elastic. Moreover, each run has been performed stimulating the skin over the non-resonant range and averaging the corresponding response.

#### 3.2.3. Indenter Selection

Two sets of tests have been performed over the same selected sensor, each set being characterized by a different indenter (radii equal to 4 mm and 10 mm, respectively). For each given indenter, the preload has been changed from a minimum (0.5 N) to a maximum (3 N) value.

**Figure 15:**
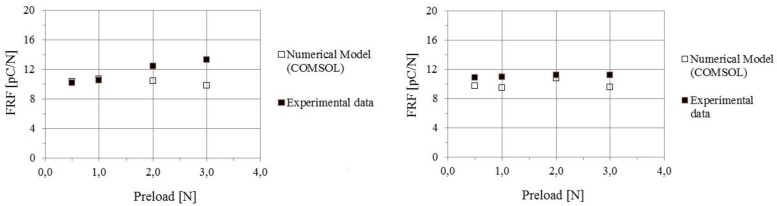
Single sensor analysis. A comparison is presented between numerical and experimental results for FRF vs. Preload, for 2 different indenters. **Left**: *R* = 4 mm, **Right**: *R* = 10 mm.

As mentioned above, comparison between experimental and numerical values of FRF (Figure 15), depends on the assumed value for d_33._ Best fit is obtained assuming that d_33_ =14 [pC/N].

Note that, with this choice, agreement is fairly satisfactory for the larger indenter, but differences are larger for the smaller indenter in the high preload range. This suggests the possibility that non-linearity of the response is responsible for such disagreement. Indeed, Figure 13 displays the presence of non-linear effects when the stress exceeds a threshold value of about 2 MPa, which is reached in the small indenter test for high preloads (recall that the effective contact radius is *a* (Equation (10))). Furthermore, non-linearity of the sensor itself may contribute. Further work is needed to investigate this issue in depth.

Anyhow, this problem has been further analyzed in the sequence of tests performed on the whole sensor array, using the small indenter (see the next section).

#### 3.2.4. Response Function of the Sensor Array

Finally, the whole sensor array has been tested, by stimulating the e-skin surface with the smaller indenter (*R* = 4 mm) aligned with the center of each selected sensor. As said in Section 3.2.2, each run has been performed over the non-resonant range at small force amplitude (F_dyn = 0.09 N) and the corresponding FRF response has been averaged over that frequency range to get a single value of the response for each sensor.

Two sets of data have been obtained. The first extensive study corresponds to higher preload (=3 N) to study the non-linearity of the whole system over a large selection of sensors (41). The second study has been performed over a smaller number of sensors (12), to measure an average response function at smaller preloads (=1 N).

Note that the given preload affects the contact radius *a* (Equation (10)) while the amplitude of the dynamic swept sine force determines the PVDF charge. On the contrary, the dynamic component does not affect the computation of the contact radius, as the dynamic signal amplitude is negligible with respect to the preload. The effective average normal stress component *T*_3_ affecting the response of the PVDF sensor area is the difference between the total stress (produced by the dynamic force amplitude added to the preload) and the stress due to the preload only. Note that—while the response of the PVDF sensor is not affected by static contacts—its piezoelectric coefficient d_33_ (Equation (15)) is likely to be dependent on the preload and some non-linearities may be expected for high preload values.

The following parameters have been used to estimate the system transfer function from the numerical model: $r_{T}$= 1.5 mm, *h* = 2.5 mm, F_dyn = 0.09 N. The radius of the Hertzian imprint has been calculated from Equation (10), using: *ν* = 0.5, *E* = 16 MPa (Section 3.2.1), *R* = 4 mm, preload = 1 N or 3 N.

However, predictions for the system transfer function depend on the effective piezoelectric coefficient d_33_ of each PVDF sensor, a quantity that was a priori unknown in the present investigation as the PDMS elastic layer was already integrated on top. Some initial characterization of the commercial PVDF film was performed around 2010 and results are reported in [21]. However, a systematic measurement of the d_33_ coefficient over the whole sensor array is not available. In any case, although such a characterization is done prior to sensor integration into the complex multilayer skin, long-term aging and fatigue may affect sensor behavior and degradation of sensor properties is expected over time. Being able to estimate the piezoelectric d_33_ coefficient from the overall system response function is thus a useful tool to measure the reliability of the e-skin device over time, whenever embedded sensors are not accessible anymore for a direct characterization.

Theoretical predictions will therefore be employed below to infer the value of the effective piezoelectric coefficient d_33_ that best fits the experimental observations.

**Figure 16:**
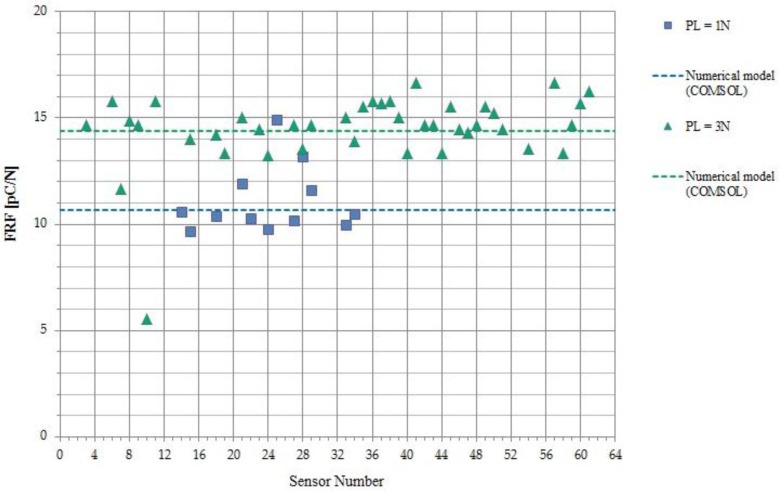
Model validation. The dynamic force amplitude is 0.09 [N] in both cases. Dotted lines correspond to the values of the response function predicted by the numerical model, for the two different preloads.

Results are reported in Figure 16.

The numerical model has been used to extract the dependence of the d_33_ coefficient, which measures the piezoelectricity of the PVDF sensor, on the preload. Data associated with the higher preload (=3 N) are well fitted with a d_33_ value equal to 22 [pC/N], while data corresponding to the lower preload (=1 N) yield a d_33_ value of 14 [pC/N]. These lower d_33_ values, associated with the latest runs, can be considered reasonable as the e-skin was subjected to a huge number of stress cycles in the past 4 years, and this has likely led to film degradation and consequent PVDF aging and fatigue. It is important to appreciate that, as already noted above, the proposed model becomes fully predictive with a preliminary measurement of the PVDF d_33_ coefficient of each sensor, prior to integration of the elastic layer on top of the sensor array.

Some dispersion of sensor response is observed, which is a measure of the accuracy of indenter positioning and of the reliability of the whole e-skin fabrication technology. Dispersion in sensor behavior may be due to various factors, which can be considered intrinsic to the manufacturing process. These factors may include different point-to-point values for the sensor radius and/or for the local layer thickness, inhomogeneity in PVDF film polarization and quality of the skin integration technology, which may affect sensor working in pure compression mode (sensor bending may occur). More sophisticated methods to precisely positioning the indenter are likely to be used in the future to further decrease the dispersion of measured sensor outputs.

## 4. Conclusions

For the considered electronic skin, contact information is transmitted through an elastomer layer to PVDF sensors that only measure pressure.

In a preliminary stage, Boussinesq’s approach has been used to solve the direct problem of how a distributed external normal force is transmitted to an extended PVDF sensor. Although this approach relies on the severe assumption to treat the elastomer as an elastic medium filling a half-space, it has allowed us to perform a comprehensive (albeit qualitative) analysis of the response of an extended sensor to a variety of forcing actions. A normal point force applied anywhere on the outer skin surface has been first investigated. The result of this study (γ curve) provides in itself a very useful practical tool for optimizing skin design to achieve a certain skin resolution. A specific validation of this analysis was not included among the scopes of the present work and will be the topic of a forthcoming publication. Next, the sensor response to a Hertzian distributed force acting on the skin surface and centered on the considered sensor has been estimated. The sensor charge response is finally written in terms of the σ parameter, which depends on the sensor and contact radii (*r_T_* and *a*, respectively), both scaled by *h*.

The half-space assumption has then been relaxed. Numerical FEM simulations have therefore been used to validate the model on a layer of finite thickness, length and width. It turned out that the numerical output follows a qualitative trend similar to that of the previous simplified analysis, but it differs quantitatively mainly due to the boundary condition imposed by the rigid substrate. However, if the substrate is moved at increasing depths higher than the depth *h* at which the sensor is embedded, then the two solutions do approach each other. This suggests that for sensors embedded into a thick protective layer Boussinesq’s analytical solution would be quite appropriate. Moreover, in the finite case, σ was found to depend on an additional parameter, L, which is a function of *R*, *F*_3_ and *E*. This parameter did not appear in Boussinesq’s analytical solution.

Finally, a real skin prototype based on a PVDF sensor array has been tested, using dynamic contacts over a non-resonant frequency range. First tests with different indenter sizes suggested the presence of some non-linear effects, attributed to both the elastomer layer and the PVDF sensor. Performing a systematic analysis over the whole sensor array, the numerical model has allowed estimation of the d_33_ coefficient, which turns out to exhibit some dependence on the preload. Fairly low d_33_ values are also found and appear to be due to PVDF aging and fatigue.

Although treated herein as a post-dictive model, the framework laid down in the present work contains all the fundamental ingredients of a fully pre-dictive model, suggesting a number of future developments potentially useful for skin design and validation of the fabrication technology. In its present form, the model is valid for incompressible protective layers (*ν* ≈ 0.5), but note that extension to compressible materials is feasible as a complete, although admittedly much more complex, solution valid for all *ν* is available [24]. On the other hand, the method is not constrained to the specific type of sensors, given that these sensors give a generic stress measurement **n**·**T** at the bottom of the elastomer layer.

Further future developments include extensions to distributed normal forces not necessarily aligned with the sensor center and to the case of multiple normal distributed loads. Modeling the effect of shear loads as well as of viscoelasticity of both the protective layer and the sensors will also deserve some attention.

## References

[1] Kim J., Lee M., Shim H.J., Ghaffari R., Cho H.R., Son D., Jung Y.H., Soh M., Choi C., Jung S. (2014). Stretchable silicon nanoribbon electronics for skin prosthesis. Nat. Commun..

[2] Hammock M.L., Chortos A., Tee B.C.-K., Tok J.B.-H., Bao Z. (2013). 25th Anniversary Article: The Evolution of Electronic Skin (E-Skin): A Brief History, Design Considerations, and Recent Progress. Adv. Mater..

[3] Tiwana M.I., Redmond S.J., Lovell N.H. (2012). A review of tactile sensing technologies with applications in biomedical engineering. Sens. Actuators A Phys..

[4] Gerratt A.P., Michaud H.O., Lacour S.P. (2015). Elastomeric Electronic Skin for Prosthetic Tactile Sensation. Adv. Funct. Mater..

[5] Tawil D.S., Rye D., Velonaki M. (2014). Interpretation of Social Touch on an Artificial Arm Covered with an EIT-based Sensitive Skin. J. Soc. Robot..

[6] Kappassov Z., Corrales J.-A., Perdereau V. (2015). Tactile sensing in dexterous robot hands—Review. Robot. Auton. Syst..

[7] Dahiya R.S., Metta G., Valle M., Sandini G. (2010). Tactile sensing—From humans to humanoids. IEEE Trans. Robot..

[8] Yousef H., Boukallel M., Althoefer K. (2011). Tactile sensing for dexterous in-hand manipulation in robotics—A review. Sens. Actuators A Phys..

[9] De Rossi D., Canepa G., Magenes G., Germagnoli F., Caiti A., Parisini T. (1993). Skin-like tactile sensor arrays for contact stress field extraction. Mater. Sci. Eng. C.

[10] Howe R.D., Cutkosky M.R. (1993). Dynamic tactile sensing: Perception of fine surface features with stress rate sensing. IEEE Trans. Robot. Autom..

[11] Shimojo M. (1997). Mechanical filtering effect of elastic cover for tactile sensor. IEEE Trans. Robot. Autom..

[12] Fearing R.S. (1990). Tactile sensing mechanisms. Int. J. Robot. Res..

[13] Fearing R.S., Hollerbach J.M. (1985). Basic solid mechanics for tactile sensing. Int. J. Robot. Res..

[14] Seminara L., Pinna L., Ibrahim A., Noli L., Caviglia S., Gastaldo P., Valle M. (2016). Towards integrating intelligence in electronic skin. Mechatronics.

[15] Seminara L., Capurro M., Valle M. (2015). Tactile data processing method for the reconstruction of contact force distributions. Mechatronics.

[16] Nalwa H.S. (1995). Ferroelectric Polymers—Chemistry, Physics and Applications.

[17] IEEE Standard on Piezoelectricity. http://blogs.cimav.edu.mx/luis.fuentes/data/files/Curso_Cristalograf%C3%ADa/piezo_ieee.pdf.

[18] Pinna L., Valle M. (2013). Charge amplifier design methodology for PVDF-based tactile sensors. J. Circuits Syst. Comput..

[19] Boussinesq J. (1885). Application des Potentielles à l’étude de l’équilibre et du Mouvement des Solides élastiques.

[20] Johnson K.L. (1985). Contact Mechanics.

[21] Seminara L., Capurro M., Cirillo P., Cannata G., Valle M. (2011). Electromechanical characterization of piezoelectric PVDF polymer films for tactile sensors in robotics applications. Sens. Actuators A Phys..

[22] Kuo Alex C.M. (1999). Poly(dimethylsiloxane). Polymer Data Handbook.

[23] Franceschi M., Seminara L., Pinna L., Dosen S., Farina D., Valle M. Preliminary evaluation of the tactile feedback system based on artificial skin and electrotactile stimulation. Proceedings of the 2015 37th Annual International Conference of the IEEE Engineering in Medicine and Biology Society (EMBC).

[24] Selvadurai A.P.S. (2001). On Boussinesq’s problem. Int. J. Eng. Sci..

